# Comparison of Curing Conditions on Physical Properties, Mechanical Strength Development, and Pore Structures of Phosphogypsum-Based Cold-Bonded Aggregates

**DOI:** 10.3390/ma17204971

**Published:** 2024-10-11

**Authors:** Guiming Wang, Zhiyi Ye, Tao Sun, Zhenlin Mo, Ziyan Wang, Gaoshang Ouyang, Juntu He, Yihua Deng

**Affiliations:** 1 School of Materials Science and Engineering, Wuhan University of Technology, Wuhan 430070, China; guimingw@hotmail.com (G.W.); zy_w@whut.edu.cn (Z.W.); oygs@whut.edu.cn (G.O.); hejt0813@163.com (J.H.); 18973455749@163.com (Y.D.); 2 International School of Materials Science and Engineering, Wuhan University of Technology, Wuhan 430070, China; yezhiyi@whut.edu.cn; 3 State Key Laboratory of Silicate Materials for Architectures, Wuhan University of Technology, Wuhan 430070, China; 4 Wuhan University of Technology Advanced Engineering Technology Research Institute of Zhongshan City, Zhongshan 528437, China; 5 Marine Building Materials and Civil Engineering Center, Science and Education Innovation Park of Wuhan University of Technology in Sanya, Sanya 572000, China; 6 China Southwest Geotechnical Investigation and Design Institute Corporation Limited, Chengdu 610052, China; 10195358@cscec.com

**Keywords:** phosphogypsum-based cold-bonded aggregates, curing condition, humidity, pore structure, specific surface area

## Abstract

This study compared the physical properties and mechanical strength development of PCBAs with water, sealed, standard, and open ambient air curing over 28 days to find a suitable curing method for the production of phosphogypsum-based cold-bonded aggregates. The types and relative amounts of hydration products, microstructural morphology and pore structure parameters were characterized utilizing XRD, TGA, FTIR, SEM and nitrogen adsorption methods. According to the results, water curing leads to rapid increases in single aggregate strength, reaching 5.26 MPa at 7 d. The standard curing condition improved the 28 d mechanical strength of the aggregates by 19.3% over others by promoting the generation of hydration products and the transformation of the C-S-H gel to a higher degree of polymerization and by optimizing the pore structure. Further, PCBAs achieved an excellent solidification of phosphorus impurities under all four curing conditions. This work provides significant guidance for selecting an optimized PCBA curing method for industrial production.

## 1. Introduction

Phosphogypsum-based cold-bonded aggregates (PCBAs) are mainly produced by the hydration reaction of phosphogypsum (PG, defined as a solid waste produced in the process of wet production of phosphoric acid) and ground granulated blast-furnace slag (GGBS, defined as a by-product with pozzolanic activity) activated by alkali-activators to generate hydration products, which encapsulate and cement the excessive PG to form cementite and produce strength [1,2]. Compared with other solid waste-based cold-bonded aggregates, PCBAs have the advantages of low production cost, a simple preparation process and broader application prospects [3]. The use of PCBAs can reduce the mining of crushed rocks, which reduces the occupation of land by PG, and the harmful impurities in PG are cured, which reduces the destruction of the ecological environment [4]. Since then, some studies have increased the PG doping to 45% or even higher to be used as road base materials, pre-products and so on, and it has been more widely used [5].

Concrete is the world’s largest volume of materials used in which the aggregate occupies up to 70% of the volume of concrete. PG can be prepared as a cold-bonded aggregate to prepare concrete with low production cost, which can reduce the use of natural coarse aggregates [6,7]. In a previous study, the preparation of PG into cold-bonded aggregates for the preparation of phosphogypsum-based concrete provided a feasible way to consume large amounts of PG [2]. The results of previous studies have shown that the 7-day compressive strength of the high-content phosphogypsum-based concretes reached 9.3–19.4 MPa, which was about 39.6% to 59.2% of the 28 d compressive strength [7]. The PCBA and concrete prepared therefrom showed great solidification capability for phosphorous and heavy metals [1,2,7].

The cold-bond function relies on hydration of GGBS activated by alkali-activators and sulfate to build cementitious properties [8,9,10,11]. Further, structures of hardened PCBAs are established by wrapping excessive PG particles with sufficient hydrates of ettringite and C-S-H gel, thus offering reliable strength [12,13]. However, due to the relatively low content of cementitious components and interference of impurity ions, PCBAs have poor early hydration properties with slow strength evolution, which shows prominent curing regime dependence, according to our previous research [1,2,7]. Due to the significant increase in the amount of PG, the hydration products become less in comparison, causing a looser structure, and the hydration reaction process in the gelling material will be more significantly affected by external conditions [14].

A notable influencing factor is the ambient curing moisture and improper curing methods, such as insufficient water, which are disadvantages to the hydration progress. Further, premature exposure to air (CO_2_) leads to the decomposition of the already formed ettringite [15,16,17]. Thereafter, a comparative study on curing conditions affecting PCBA property evolution is desired so as to guide combined curing method selection for optimizing PCBA production.

PCBAs successfully achieve the large-scale consumption of PG and make contributions to alleviating the shortage of natural aggregate. Compared with conventional sintered aggregates, the production and employment of PCBAs characterize low-cost, low-CO_2_ emission with far less energy demand. Moreover, unlike natural aggregates whose properties remain constant, the structure and properties of PCBAs change gradually as hydration proceeds. Ettringite is a primary hydration product of PCBAs, whose production and crystal growth are affected by internal factors such as ion concentration in pore solution (i.e., sulfate, aluminates, alkaline ions, etc.), as well as the external environment, such as humidity [9,18,19]. Moreover, it has been reported that the hydration process of cementitious materials varies under different curing methods [20,21,22] in which water is present in different forms in the material before and after hardening [8,23]. During the early stage of the hydration reaction, multiple ions need to enter the aqueous solution in order to react; then, water is physically and chemically bonded to the hydration products of ettringite and C-S-H gel [9,12,24]. The excess water exist in the form of free water in the capillaries [25]. As far as PCBAs are concerned, the ratio of water-to-powder during granulation is limited to 0.14–0.16, which indicates a very low water content level. However, the water content in the aggregates (including the internal water and the water in the environment) plays a significant role in strength development during hydration.

Another important aspect of PCBAs is the pore structure as a result of the pelletization process, powder particle size, water evolution and spatial accumulation of hydration products, which is also influenced by the curing conditions at the initial stage of hydration [20,26]. It is believed that the internal pore structure is responsible for the macroscopic properties and mechanical characteristics of hardened materials [11]. As an example, pores can act as channels of moisture transport and CO_2_ diffusion, and the microstructure evolution due to hydrate accumulation and carbonation can also affect pore structure, thereby impacting the macro performance [15].

For the curing condition, Tajra [27] et al. suggested that relative humidity significantly affects the mechanical strength of aggregates, and that core–shell aggregates produce the fastest strength development at 99% RH. Manikandan et al. [19] carried out a series of studies on the modes of maintenance of aggregates and investigated the rate of hydration reaction of the aggregates and found that both autoclave and steam curing significantly reduced the time of curing. The mechanical strength of lightweight aggregates cured in steam for 10 h can increase rapidly for a short period of time up to more than 85% of the strength of 28 d water curing. It can be concluded that the effect of humidity on the mechanical properties of cold-bonded aggregates is significant. Particularly, industrial-scale aggregate production often requires lower cost and simple process, with the same for storage conditions. From a comprehensive consideration of convenience and cost, the optional curing methods include sealed curing, water curing, standard curing and natural curing. Sealed curing completely isolates the aggregate from the surrounding environment but may be difficult to implement. Curing with water can facilitate strength growth but it may cause potential pollution of the water because of the phosphorus impurities [28]. For standard curing, it is necessary to maintain a strict temperature and humidity control environment. Natural curing is the simplest and most economical curing option but the low alkali content easily leads to carbonation, which is unpleasant for stable ettringite growth, despite there some research indicating that using accelerated CO_2_ curing for cold-bonded aggregates can sequestrate 3.5–4.1 wt.% CO_2_ and the produced environmentally friendly by-products [29]. Furthermore, consideration should be given to the changes in the structure and properties of PCBAs under natural curing conditions.

Therefore, this paper focused on the effects of four common curing conditions, including water, sealed, natural and standard curing on the macroscopic physical and mechanical properties of PCBAs, as well as the microstructural evolution. SEM, XRD, TGA, FTIR and low-temperature gas adsorption methods were used to characterize the hydration products and pore structure of PCBAs. Furthermore, the phosphorus leaching of PCBAs was assessed since PG possessed potential phosphorus leaching pollution [28]. It is the first time that a large amount of PCBA is prepared using disk pelletizing technology and placed in four different curing environments to study the relationship between its macroscopic mechanical properties and microstructure. The findings of this are expected to solve the problem of the lack of suitable curing conditions and curing time for industrial production and provide an economic guarantee for industrial production. 

## 2. Raw Materials and Methods

**Figure 1:**
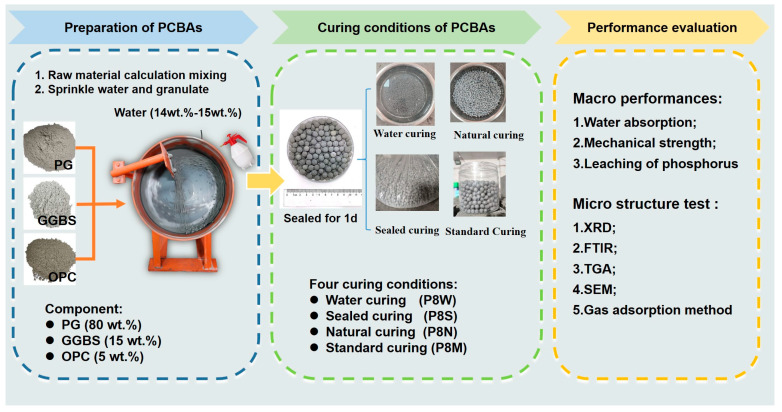
Experimental procedures in this study.

The experimental procedures of this study are shown in Figure 1. In this study, PCBA was prepared and placed in four different curing conditions to evaluate the physical and mechanical properties. 

### 2.1. Raw Materials

**Figure 2:**
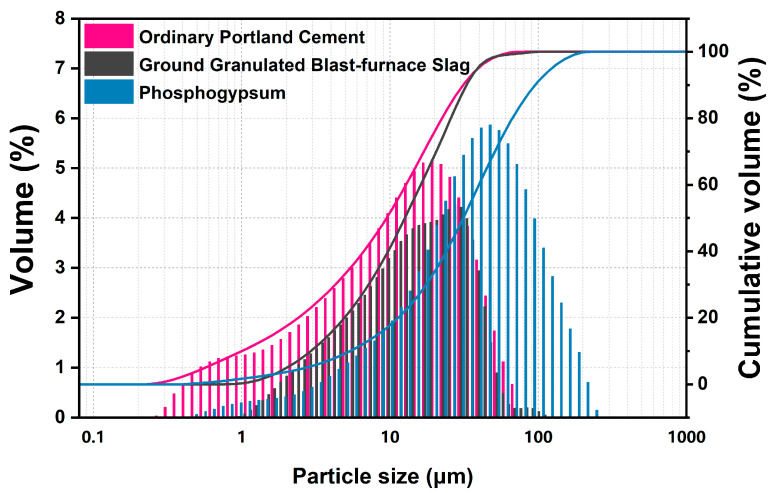
Particle size distribution of phosphogypsum, ordinary Portland cement and ground granulated blast-furnace slag.

Fresh original phosphogypsum was dried at 40 °C for more than 24 h and then sieved through a sieve with 0.6 mm square hole, obtaining PG powder with an appropriate particle size for further hydration (D_50_ = 33.87 μm). The ordinary Portland cement and GGBS were produced by Huaxin Cement Co., Ltd. (Huangshi, China) and Hubei Xinye Steel Co., Ltd. (Huangshi, China). The chemical composition of three raw materials was determined by XRF, and the physical performances also were evaluated in Table 1 and Figure 2. Phosphogypsum is mainly composed of 81.59 wt.% gypsum per SO_3_ content, small amount of quartz and phosphorus. 

According to GB/T 18046-2017 [30], the hydration reactivity of GGBS was evaluated with an actual activity index, as well as mass coefficient calculated as Equation (1) [2]. The activity index of slag reached 86% and 99% at 7 d and 28 d, respectively, exceeding the requirements of the national standard of 70% and 95%. Additionally, the calculated *K* with value of 2.04 means that GGBS used in this study possessed high activity and contributed to the persistent hydration of PCBAs.

(1) $$K=\frac{m_{\mathrm{CaO}}+m_{\mathrm{MgO}}+m_{{\mathrm{Al}}_{2}O_{3}}}{m_{{\mathrm{SiO}}_{2}}+m_{\mathrm{MnO}}+m_{\mathrm{Ti}O_{2}}}$$

### 2.2. Mix Proportion and Preparation Process

There has been some discussion [2] regarding PCBAs with PG proportions between 60 wt.% and 80 wt.% exhibiting satisfactory macro performance, in which the bulk density, water absorption, compression strength and softening coefficient meet the specifications of GB/T 17431.1 [31]. As a result of the significant reduction in degree of hydration, physical and mechanical performance deteriorates rapidly with the further incorporation of PG. To ensure the strength development and durability of PCBAs in this study, the PG, GGBS and cement content are restricted to 80 wt.%, 15 wt.% and 5 wt.%.

In this study, PCBAs were prepared using the stirring granulation method, divided into two steps. Firstly, the process of “nucleation”, which affected the whole granulation process as well as the performances of aggregate, was the most important step. Another characteristic was the growth of aggregate balls, during which the aggregate had formed preliminarily. Next, the coated powder and the initial PCBA balls were fused into one as the aggregate grew under the influence of adhesive water. The whole granulation process was carried out in a disk granulator with an outer diameter of 50 cm, an inner diameter of 49 cm, a depth of 16 cm, a fixed inclination of 45° and a speed of 43 r/min and kept constant.

Phosphogypsum, ground granulated blast-furnace slag and cement were weighed according to 80:15:5 mass ratio in the disk granulator and mixed evenly. The preparation of PCBAs is divided into two steps:

1. Seeding. Take 200 g of the mixed powder and pour it into the uniformly rotating disk granulator; use the atomizing spray can to spray a small amount of water to make the powder in the disk slightly wet. After all the powder is wet, continue to add the power mixture; the wet powder in the disk will agglomerate to form the seed pellets. Alternate steps of adding powder and sprinkling water. When the seed pellets are still fragile, a rolling and curtain-dropping movement will make them constantly collide with the force squeeze to become dense.
2. Size enlargement. After the formation of seeds, a small amount of atomized water is sprayed into the disk to moisten the surface of these microspheres. Then, a small amount of powder is evenly sprinkled into the disk to make the newly added powder evenly adhere to the surface of seeds, and the powder is fused with the aggregate microspheres under the effect of water adhesion. Then, under the action of gravity, the pellets fall along the arc of the disk surface to the bottom of the pelletizing disk. During the movement, the pellets are continuously compacted from the inside out by the compression of the inner wall of the pelletizing disk and by their own gravity. The process is repeated several times until the pellet diameter increases to 8 mm. The size is controlled by sieving.

The amount of water used for granulation is 14–15 wt.% of the powder mass. The formed fresh pellets were transferred to sealed containers for 1 d at room temperature, and then placed in natural condition, sealed condition, water immersion condition and standard condition. 

The characteristic parameters of four curing conditions are listed in Table 2. Natural curing was carried out with the aggregate in an ambient air at 20 ± 2 °C indoors, named P8N. Water curing was the curing of the aggregate by immersing it in water at 20 ± 2 °C, named P8W. Seal curing was the plastic wrapped against air and curing at 20 ± 2 °C, named P8S. Standard curing was carried out in a permeable mesh bag and placed in a standard curing room at a temperature of 20 ± 2 °C and a relative humidity of 90% or more, named P8M. In this paper, the aggregate was cured in a permeable mesh bag and placed in a standard curing room. In order to ensure the same and constant temperature, we used air conditioning to control the temperature.

### 2.3. Testing Methods

**Figure 3:**
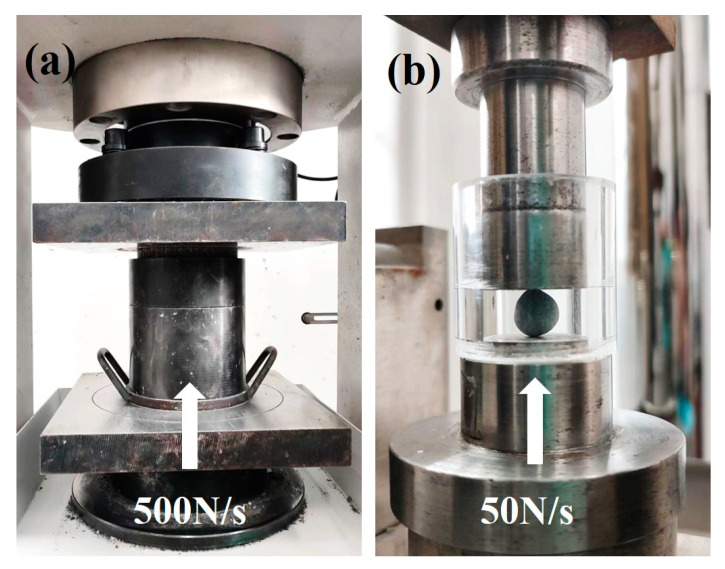
Testing methods of mechanical strength: (**a**) cylinder compressive strength; (**b**) single pellet compressive strength.

The bulk density, water absorption, cylinder compressive strength and softening coefficient of the aggregates were tested according to national standard GB/T 17431.2 [32]. It was recommended that PCBAs should be dried at 45 °C before testing in order to attain a constant weight. The testing method of cylinder compressive strength is shown in Figure 3a.

The aggregates used in the single aggregate strength test are small spherical aggregates with a diameter of 8 mm. Single aggregate strength was obtained by a measurement of the crushing load of aggregates shown in Figure 3b. It was recommended to record the maximum value of the aggregate when it was damaged by the pressure load as 50 N/s between parallel plates. The measurement should be repeated for more than twenty samples, and the average should be taken. According to Equation (2), the strength of a single aggregate was introduced [33]:

(2) $$\sigma =\frac{2.8\times P}{\pi \times d^{2}}$$

where *d* was the diameter of aggregates (mm), *P* was the broken force when aggregates were crushed (N).

### 2.4. Microstructure Testing Scheme

Prior to the microstructure and phase tests, PCBAs cured for 28 d were crushed to a size less than 75 μm and dried under vacuum at 40 °C for 24 h. Sample preparation should be rapid and prevent carbonation. The samples were measured with an Empyrean X-ray diffractometer with a copper target X-ray tube and operated at 40 mA and 40 kV. X-ray pattern was collected over a range of 5° to 70°. 

The Thermogravimetric Analysis (TGA) spectra recorded at Netzsch STA449F3 (Selb, Germany) were monitored in the temperature range of 25 to 900 °C with a heating speed of 10 °C/min under a 20 mL/min flow of N_2_. 

A mixture of sample powder and potassium bromide was pressed into tablets at a ratio of 1:100 using a Fourier transform infrared reflection (FTIR) instrument (Nexus spectrometer, Therno Nicolet, Madison, WI, USA.) in the mid-infrared region from 4000 cm^−1^ to 400 cm^−1^. The microstructure of the material at the micron level can be observed with scanning electron microscope (SEM), which enables the structure and morphological distribution of the crystals to be determined more accurately. The microstructure of PCBAs was examined using a Zeiss Ultra Plus scanning electron microscope (Oberkochen, Germany) operating at low vacuum and 10–15 kV accelerating voltage. The SEM had a resolution of 1.0 nm with a magnification of 12–100,000×. Due to the poor electrical conductivity of PCBAs, the sample surfaces were plated with platinum (Pt) in vacuum for 600 s before the test.

Gas adsorption was a common method for determining the specific surface area and pore structure of porous materials [34,35,36]. The ASAP 2460 specific surface and pore volume analyzer (ASAP 2460, Micromeritics, Atlanta, GA, USA) was used for the low-temperature nitrogen adsorption experiments, with a pore size range of 0.4–200 nm and a minimum pore volume of 0.0001 mL/g. Samples were first degassed by vacuum drying at 40 °C for 24 h in a vacuum oven at a vacuum level of 0.06 MPa, and then 2.0 g of the sample was taken as adsorbent with high-purity nitrogen and the amount of nitrogen adsorbed at 77 K at different relative pressures p/p_0_ was measured. The adsorption/desorption isotherm was plotted using the relative pressure p/p_0_ as the horizontal coordinate and the adsorption amount per unit mass of sample as the vertical coordinate. The specific surface area and the total pore volume of the sample were determined based on the BET equation using the adsorption branch as the data source. The pore size distribution was calculated using the BJH (Barrett–Joyner–Halenda) model based on the Kelvin equation with the desorption branch as the data source.

### 2.5. Phosphorus Leaching Test

#### 2.5.1. Static Leaching Test

The PCBAs cured for 28 d were vacuum-dried at 45 °C for 24 h, about 100 g of PCBAs was collected and mixed with deionized water at a ratio of 5 L/kg and left at room temperature (23 ± 2 °C) for 28 d [2]. The soluble phosphorus content of PCBAs and original PG was measured with an ultraviolet spectrophotometer after color development. 

#### 2.5.2. Acetic Acid Leaching Test

The acetic acid leaching test was leached using the horizontal vibration method with reference to standard HJ 557-2010 [37]. The PCBAs and fresh PG were crushed, and a mixture of 20 L/kg of acetic acid was placed in a conical flask. About 10 g of PCBA particles were collected after grinding and crushing in the mortar. The leachate was filtered through a 0.45 μm microporous membrane and the phosphorus content was measured with an ultraviolet spectrophotometer after digestion and color development.

## 3. Results and Discussion

### 3.1. Physical Properties

#### 3.1.1. Bulk Density

**Figure 4:**
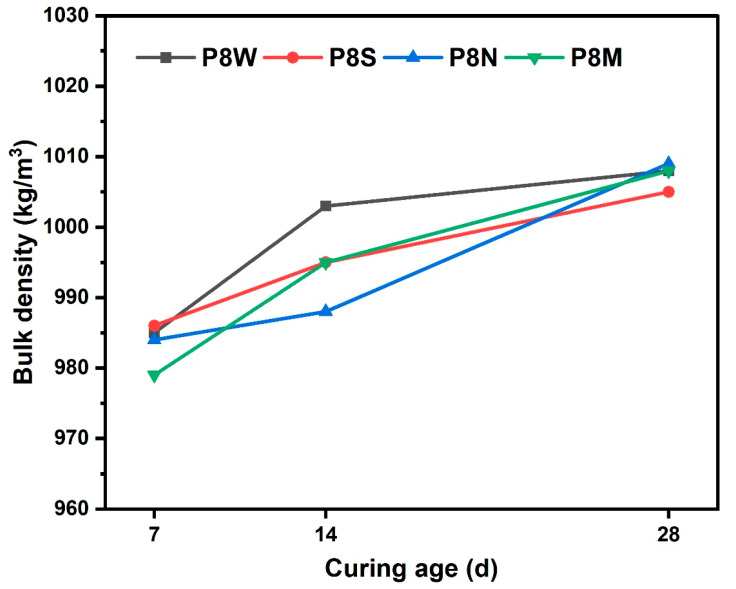
Bulk density of PCBAs at 7 d, 14 d and 28 d.

The bulk density of PCBAs at 7 d, 14 d and 28 d under different curing conditions is shown in Figure 4. The bulk density of the dried PCBAs prepared by cold bonding technology is less than 1100 kg/m^3^, which meets the requirement of GB/T 17431.1 that the density of lightweight aggregates should not exceed 1200 kg/m^3^. Density increases with curing age, hydration products increase, densification increases and density increases. There is no significant difference in bulk density between various curing conditions.

#### 3.1.2. Water Absorption 

**Figure 5:**
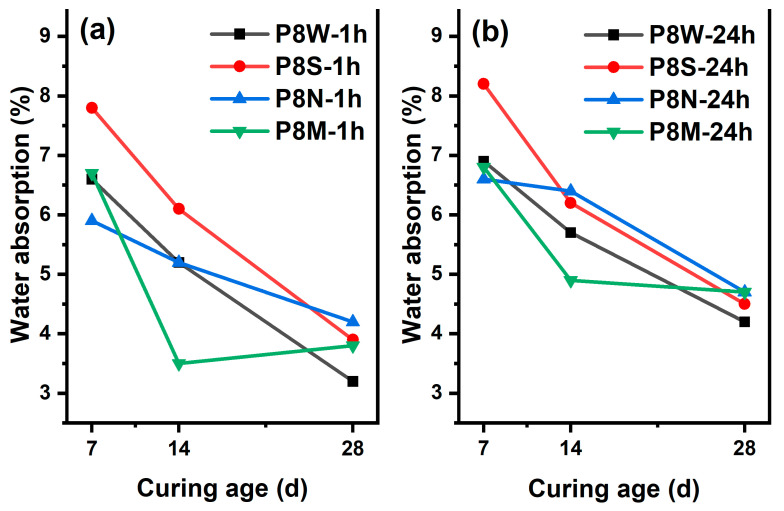
Water absorption rates at 7 d, 14 d and 28 d: (**a**) 1 h; (**b**) 24 h.

As a critical indicator of the performance of manufactured aggregates, the 1 h and 24 h water absorption rates of PCBAs at 7 d, 14 d and 28 d under the four curing conditions are evaluated, as Figure 5 shows. A declining trend in the 1 h and 24 h water absorption rates is noticed in all samples with the prolonging of curing age, which even reaches 7.8% and 8.2% in the PCBAs with sealed curing at 7 d, indicating that the low ambient humidity is not favorable for hydration hardening and structural development. Furthermore, the 1 h and 24 h water absorption rates are the lowest in the P8S after standard curing for 14 d, at 3.5% and 4.9%, respectively. In contrast, the other samples display similar levels of 24 h water absorption. A similar drop in water absorption was observed between 4.2% and 4.7% for all four PCBAs at 28 d. The lowest water absorption at 7 d in P8N and the highest at 28 d indicates that carbonation leads to the decomposition of the hydration products [15,38,39], leading to an increase in the pore structure and an increase in water absorption; both 1 h and 24 h water absorption is the lowest at 14 d. 

Considering the similar absorption behavior of P8W, P8N and P8M at 7 d, it can be assumed that the standard curing conditions are the most suitable for hydration hardening and the fastest structural development of the PCBAs. There were no aggregates that exceeded the 10% limit for 1 h water absorption of aggregates set by GB/T 17431.1. The water absorption of aggregates gradually decreases with an increase in the curing period as a result of hydration reactions within the aggregates [40]. As the GGBS gradually dissolves, the generation of hydration products precipitates and optimizes the pores, increasing the density of the pastes [1].

### 3.2. Mechanical Properties

#### 3.2.1. Cylinder Compressive Strength and Softening Coefficient

As seen in Table 3, the cylinder compressive strength of the PCBAs differed significantly with regard to the curing conditions. Apart from the P8N under natural curing, all PCBAs meet the requirements of GB/T 17431.1-2010 [31], with cylinder compressive strengths exceeding 6.5 MPa. The prepared PCBAs are far stronger than the limited value of 5–6.5 MPa in GB/T 17431.1–2010 [31] and slightly higher than other industrial solid waste cold-bond aggregates (4.5 MPa to 12.59 MPa) that were reported in Refs. [11,41]. Both standard curing and water curing are conducive to the development of their 28 d dry cylinder compressive strength, which reaches 11.3 MPa in the sample under water curing. A significant contribution to its hydration reaction may be due to the presence of sufficient water in the curing environment. PCBAs with a natural curing condition had a cylinder compressive strength of 7.2 MPa, which was lower than that for PCBAs with a sealed curing condition of 8.6 MPa, as well as the development in the softening coefficient. 

In comparison, the development of PCBA cylinder compressive strength was more favorable when water curing was used as opposed to standard curing. A sealed curing condition led to slower PCBA strength development, while a natural curing condition led to unfavorable results attributed to water in the aggregate evaporating with time, which affects the hydration process, and some alkaline substances in the system were carbonated.

#### 3.2.2. Single Aggregate Strength

**Figure 6:**
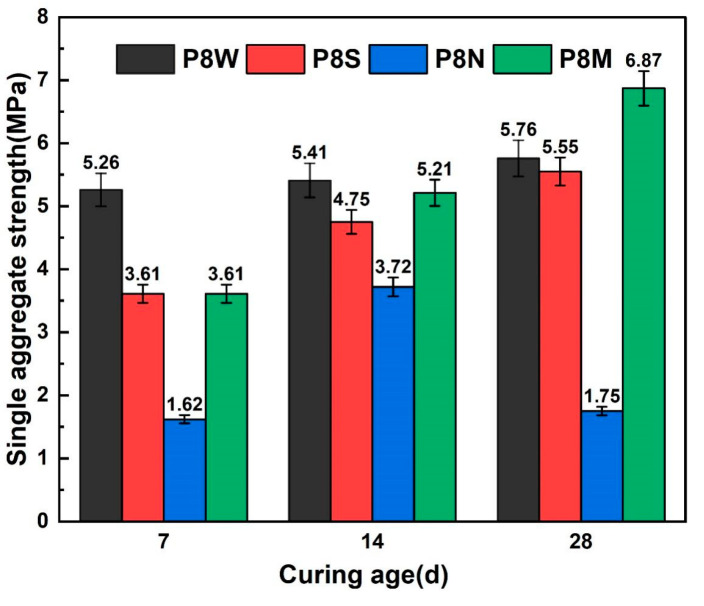
Single-particle strength at 7 d, 14 d and 28 d.

Figure 6 illustrates the single-particle strength of PCBAs. Increasing the curing age had a positive effect on the single-particle strength of the aggregates. As early as 7 days after the aggregates were cured, the single-particle strength of the water-cured aggregates developed the fastest. However, a negligible difference is detected between the single-particle strength for aggregates under standard and sealed curing conditions. Comparing the strength growth of PCBAs under the three curing conditions, a relatively high strength of 5.26 MPa is achieved at 7 d in the water curing, followed by very slow strength development at 14 d and 28 d. The single-particle strength for the P8S under sealed curing reaches 3.61 MPa at 7 d, and the 14 d-strength increase ratio is greater than that during the later age. For the aggregates cured under standard conditions, the single aggregate strength at 7 d and 28 d reaches 3.61 MPa and 6.87 MPa, respectively, indicating that this curing method is capable of showing an excellent effect on potential strength development compared to other methods. In contrast, the naturally cured samples possess the lowest strength at 7 d despite growing until curing for 14 d, displaying a sharp inversion at 28 d. 

For the single strength of aggregates cured for 28 d, standard curing increased by 23.8% over the sealed curing condition, 19.3% over the submerged curing condition, and 292.6% over natural curing. For 28 d dry cylinder compression strength, standard cured aggregates showed an increase of 40.3% over natural curing. 

**Figure 7:**
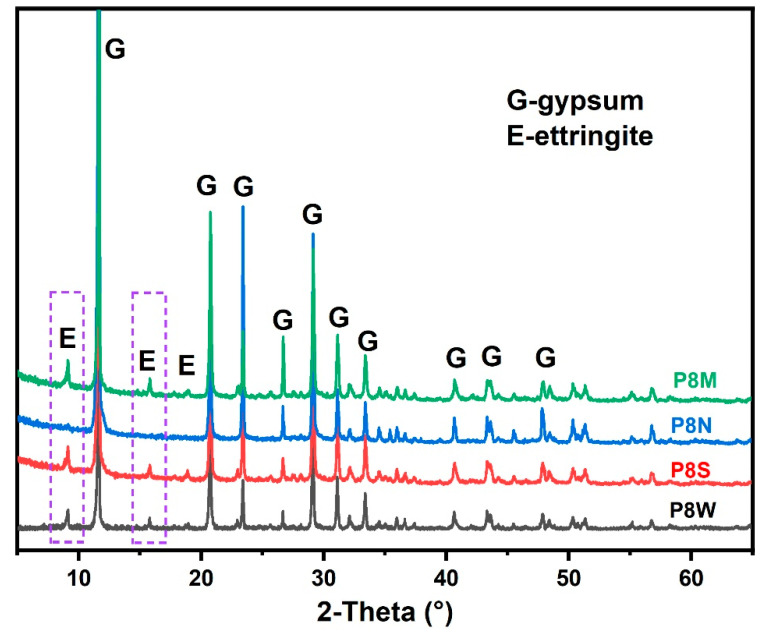
X-ray diffraction (XRD) spectra of phosphogypsum-based cold-bonded aggregates at 28 d.

Based on the phase assemblage analysis in Figure 7 and Table 4, it is clear that the hydration products, including ettringite (marked in the purple dotted box) and C-S-H gel, which provide the cementitious ability, have essentially become completely carbonated and experienced shrinkage, which explains the decrease in mechanical strength [15,42,43,44,45].

In a comprehensive analysis of the single aggregate strength with age for each group of PCBAs, it was found that supplemental water provided at an early stage favors their strength development, whereas too much water will result in a reduction in later strength development. Although the strength of aggregates develops rapidly under the condition of water curing, the potential leaching may be the reason for the limited development of mechanical properties in the later stage. Some studies have pointed out that the shrinkage caused by the early sealed curing may be the reason for the deterioration of the mechanical properties of aggregates [46]. Standard curing creates an appropriate humidity without leaching, of which the 28 d-strength is significantly enhanced over those of the other curing methods. Therefore, the effect of various curing methods on the generation of hydration products and microstructure construction are investigated further to determine the water migration and binding mechanism. 

### 3.3. Hydration Products and Microstructure Analysis 

#### 3.3.1. Hydration Products

The X-ray diffractometer patterns of PCBAs at 28 d are detailed in Figure 7. The characteristic peaks at 9.1° for ettringite and 11.6° for gypsum dihydrate can be observed from the plots. It is noteworthy that the diffraction peak for portlandite is also not observed, as the portlandite would be consumed to produce ettringite due to the excess gypsum [2,41,47]. In general, the strong diffraction peaks at low angles are important for reference purposes. 

Compared to P8S and P8W, the relative peak intensity (ettringite/gypsum) of P8M is the highest, which means the highest hydration degree. After 28 d of natural curing, no characteristic diffraction peaks are seen for ettringite, nor for calcite, which reacts with water and CO_2_ in the air to form indeterminate calcite and secondary gypsum [15]. This is confirmed by the TG curves that follow. 

**Figure 8:**
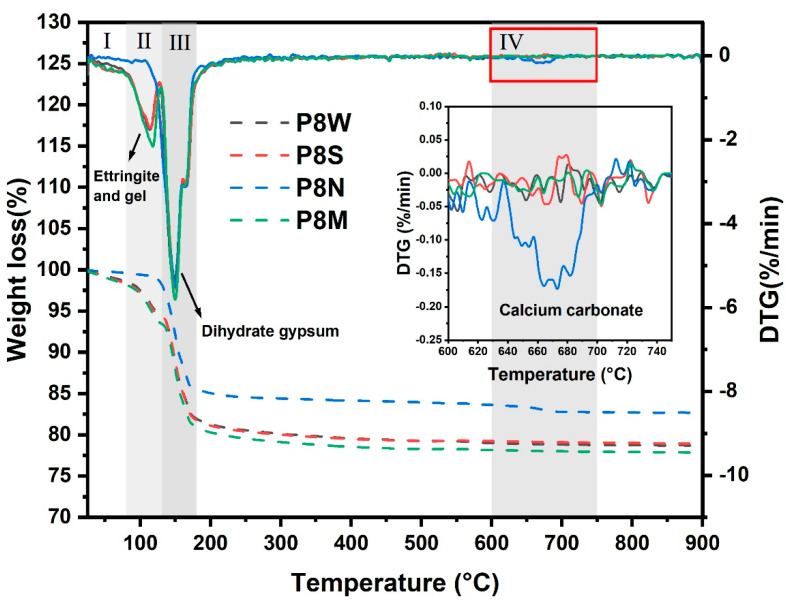
TGA curves of phosphogypsum-based cold-bonded aggregates at 28 d under four curing conditions.

The TGA curves of the aggregates at 28 d for the four curing conditions are shown in Figure 8. As the temperature increases, several substances undergo thermal decomposition, as follows: (1) the removal of adsorbed water from the C-S-H gel interface [12,48,49] and part of structural water of ettringite within 80–130 °C [12,50]. (2) The dehydration of gypsum dihydrate is determined in all samples in the thermal range of 130–180 °C, which presents a strong weight loss at the TG curves. (3) Furthermore, PCBAs cured under natural curing exhibit a very slight weight loss when heated to 620 °C, which is the typical decomposition temperature of calcium carbonate [15,43]. Based on this, the TGA curve was segmented into four temperature intervals. The first interval is 25–80 °C, the second is 80–130 °C, the third is 130–180 °C, and the fourth is 600–750 °C.

It can be concluded from the TG curves that the amount of weight loss (mainly caused by the loss of physically or chemically bound water) is the highest in the standard-cured sample compared to the sealed-cured and water-cured samples, where the fastest decomposition rate was noticed within the first period. There is a slight discrepancy in thermal decomposition rates in each period between the samples under these two curing conditions on the DTG curves, as well as the total weight loss. Particularly, a weak thermal decomposition peak associated with the water loss of hydrates is detected in the DTG curve of P8N, where the mass loss is concentrated in the range of dehydrated gypsum decomposition.

Powers et al. [51] pointed out that hardened cement pastes consist of hydration products, capillary water and unhydrated cement, where the hydration products contain some water. The capillary water is the physically bound water inside the pores and was removed before the TGA test. Therefore, the water content in hydration products can be considered an indicator of the degree of hydration reaction [48], which is consistent with the trend of the single aggregate strength of PCBAs at 28 d. 

Considering that cement and GGBS have hydrated completely with less of an influence on mass loss, we take the total weight loss at 750 °C as a reference, where the mass loss related to different substance decomposition is calculated and summarized in Table 4. In this experiment, the interval 45–750 °C was chosen to calculate the total weight loss, W_Total_; the weight loss by thermal dehydration of excess gypsum dihydrate between 130 and 180 °C, W_g_; and the water content of the hydration product, W_H_ = W_Total_ − W_g_. For P8N, there was no longer a weight change in zones I and II, and the mass loss in zone IV was calculated to be 2.818 wt.% calcium carbonate.

The weight loss of water in hydration products reaches 4.471 wt.% in P8M, with an increased ratio of 29.71% and 42.75% compared to P8W and P8S, respectively. Correspondingly, the weight loss of dihydrated gypsum shows a declining trend with the consumption for the generation of ettringite. This implies that standard curing conditions with suitable temperature and humidity contribute to hydration, leading to continuous strength development. After 28 d of natural curing, there is basically no water mass loss related to the hydration product, while the mass loss attributed to the carbonate thermal decomposition reaches 2.818%. Combined with XRD analysis, the calcium carbonate produced by the carbonation of PCBAs under natural curing conditions is aragonite, with a low degree of crystallization. The complete carbonization of the hydration products is the main reason for their low mechanical strength. In the absence of water during the hydration process of GGBS, the release of ions and dissolution of ions in the pore solution are affected, leading to a relatively lower degree of hydration. By combining with alkaline substances, the erosive CO_2_ will weaken the pH value and may even result in the degradation of hydrates [15,43,44].

**Figure 9:**
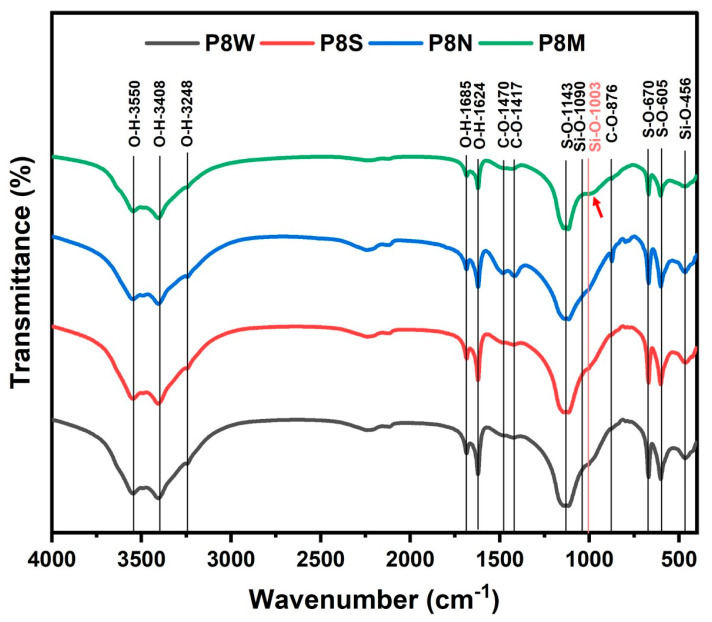
Fourier transform infrared reflection spectra of phosphogypsum-based cold-bonded aggregates at 28 d under four curing conditions.

Figure 9 describes the FTIR analysis profile of PCBAs at 28 d under different curing conditions, and the characteristic wavenumbers of the bands are listed in Table 5. The analysis of the FTIR spectra is based on the Lambert–Beer law, where multiple absorption peaks may usually overlap each other, and therefore, the structural characteristics of the phases need to be analyzed by the location of the number of characteristic absorption [52]. The bands located at 3550 cm^−1^, 3408 cm^−1^, 1685 cm^−1^ and 1624 cm^−1^ are ascribed to O-H vibrations in the structural and free water in hydrates and gypsum [15,23]. The strongest absorption of O-H vibration is observed in the water-cured sample. 

Combined with the thermal analysis, it is suggested that more water is absorbed in the PCBAs as free water during the water curing condition. The S-O bending vibrations in ettringite and gypsum are noticed at 605 cm^−1^ and 670 cm^−1^, in addition to S-O symmetric stretching vibrations of SO_4_^2−^ tetrahedra within 1100–1200 cm^−1^. The C-O bands found near 1400 cm^−1^ and 876 cm^−1^, the absorption intensity of which presents stronger and sharper in P8N, indicates a larger amount of carbonates [55,61]. Compared to other samples, the characteristic band position of the Q^2^(Si-O) symmetric stretching vibration within 970–990 cm^−1^ in the standard-cured sample shifts towards a larger wavenumber and a shoulder at 1003 cm^−1^, indicating a generation of C-S-H gel with a higher polymerization degree [52,57,62]. The starting point of the shoulder at 943 cm^−1^ for PCBAs may be assigned to Si-O^-^ terminal bonds, which means a higher Ca/Si ratio of C-S-H [63]. The optimization of the structure of the gel contributes to the construction of cementitious structure, enhancing the macro performances, which are determined by the strength test results (Figure 6).

**Figure 10:**
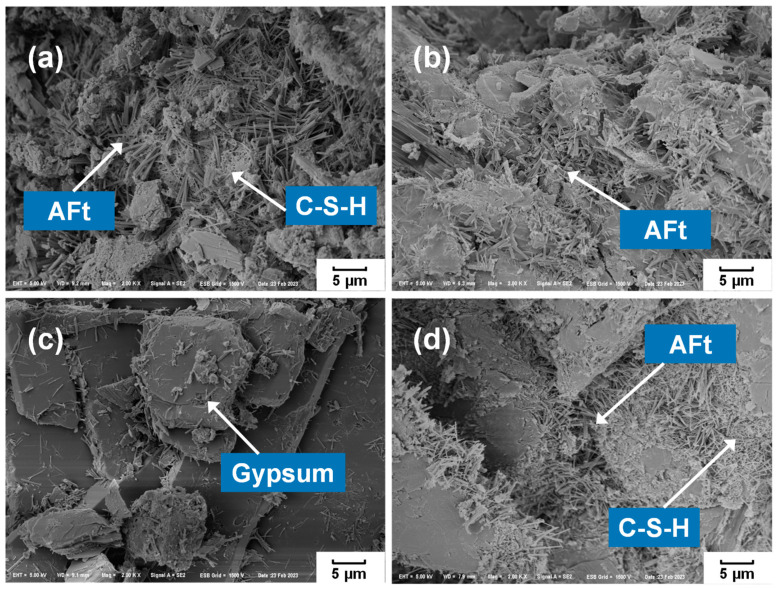
Microstructure of phosphogypsum-based cold-bonded aggregates at 28 d: (**a**) water cured; (**b**) seal cured; (**c**) natural cured; (**d**) standard cured.

In Figure 10, the microstructure of PCBAs can be clearly seen, where a number of needle-rods of ettringite and C-S-H gels with a porous honeycomb cluster generated and gathered in the holes and cracks [2,64,65]. The P8M group exhibited a greater number of elongated needle-rods of ettringite and C-S-H gels with porous honeycomb clusters growing between the excess gypsum crystals, which was corroborated with the TGA results. Especially, there is little ettringite and gels in the P8N, resulting in a weak cementitious ability for the subsequent microstructure development.

The dissolution of GGBS activated by sulfate and alkaline hydrates originating from cement hydration has provided sufficient active groups for further polymerization and precipitation of cementitious phases. As a result of the relatively high level of PG in hardening pastes, an adhesive bond is established between unreacted particles instead of a complete encapsulation of the particles. As an important medium for ion dissolution and transfer, water’s content and exchange rate with the external environment will affect the formation of hydration products, as well as pore structure and distribution by affecting the ion concentration and transport rate within the local pore solution.

Where cement and GGBS are cementitious materials in the aggregate, cement takes the lead in the hydration reaction in the presence of water to produce hydration products such as portlandite, providing the alkaline conditions to excite the hydration of GGBS under the combined action of gypsum to form more ettringite and C-S-H gels [2,7,29,47]. As the hydration reaction proceeds, C-S-H gel and ettringite are produced and the PCBAs become denser, resulting in increasing strength and density decreasing in water absorption [2]. Compared to other PCBAs, P8N shows a significant reduction in cold-bonded mechanical strength and physical properties.

#### 3.3.2. Specific Surface Area and Pore Size Distribution

**Figure 11:**
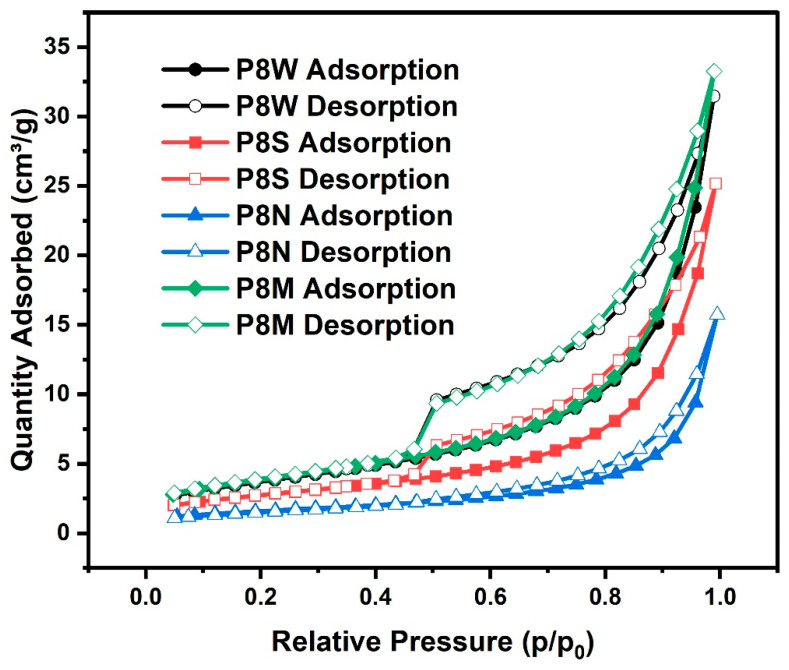
Isotherms of the phosphogypsum-based cold-bonded aggregates at 28 d under four curing conditions.

The isotherms of all samples are depicted in Figure 11. As the relative pressure increases, the main processes of nitrogen adsorption are monolayer adsorption, multilayer adsorption and capillary coalescence in the order of pore filling, corresponding to micro-pores (<2 nm), meso-pores (2~50 nm) and, finally, macro-pores (>50 nm), and the desorption is reversed. The shape of the isotherms reflects pore structure characteristics, such as pore shape parameters and total pore volume of the sample, etc. [34,66,67]. It is important to realize that most of the isotherms are not defined by IUPAC as usual isotherms, which means they are generally a combination of two or more different types of isotherms in reality [68]. 

Comparing the isotherms of PCBAs in the four curing conditions, the curves almost overlap when the relative pressure p/p_0_ is less than 0.4. This indicates that there are a small number of microporous holes (<2 nm) in the pastes, such as cylindrical pores closed at one end, slit pores in parallel plates or ink bottle pores [36]. It is not evident that the microporous hole content varied between the samples cured in the different conditions. When a relative pressure of p/p_0_ exceeds 0.5, the isotherms separate and form a hysteresis loop whose shape and slope are dependent on the condition. It has been noticed that the hysteresis loop area of P8M is slightly larger than that of P8W and P8M. All of the inception points of these hysteresis loops are located approximately at a middle relative pressure of 0.50, except for P8N. In other words, there is no abrupt mass desorption process, and the distribution of pore sizes becomes more evenly dispersed. The area of the hysteresis loop of the P8M group was slightly larger than that of P8W and larger than that of P8S, indicating an increasing amount of C-S-H with a higher Ca/Si ratio. Compared with P8W and P8S, P8M possesses a slightly larger hysteresis loop area, indicating the production of C-S-H gels with a higher Ca/Si ratio [68]. Particularly, a plurality of middle meso-pores predominantly existed in C-S-H gels and was shown to be more significantly responsible for the adsorption of gas in PCBAs.

Table 6 illustrates the specific surface area of PCBAs under four curing conditions calculated by the BET approaches. PCBAs cured in standard and water condition possess greater specific surface area, reaching 13.83 m^2^/g and 13.38 m^2^/g, respectively. This means that sufficient water can promote the hydration degree of pastes, while excessive water will later lead to a slow hydration reaction. 

The water and standard curing conditions are beneficial to the hydration reaction, with the largest specific surface area, followed by water cured and 13.38 m^2^/g under sealed curing. Lack of water under sealed conditions and without external moisture supplementation results in a slower hydration process, producing a smaller specific surface area than the P8M and P8W groups. 

**Figure 12:**
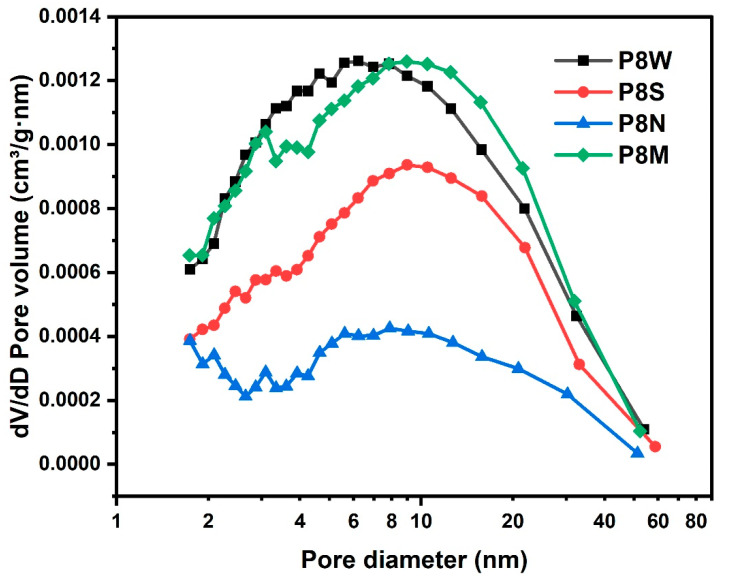
Pore size distribution of the phosphogypsum-based cold-bonded aggregates at 28 d under four curing conditions.

The pore size distribution of the PCBAs is shown in Figure 12. Natural curing showed wide pore size distribution. The same pattern was observed for the average pore size, with the standard and water-cured PCBAs having a smaller average pore size of 8.9 nm and 8.5 nm, respectively, and a denser structure. For the natural cured aggregates, the average pore size reaches 15.8 nm, the pore size distribution becomes dispersed and the number of large pores increases due to the continuous carbonation and decomposition of ettringite and the gradual decalcification of the C-S-H gel to form a low Ca/Si C-S-H gel and the coarsening of the pores [38,43,69]. 

### 3.4. Leaching Behavior of Phosphorus

The leaching behavior of phosphorus is evaluated for the environmental impact assessment, as detailed in Table 7. In comparison with fresh PG, PCBAs under four curing conditions achieve an excellent solidification of phosphorus impurities. Due to the alkaline environment in PCBAs, phosphorus elements are fixed in the form of calcium phosphate and calcium hydrogen phosphate [70], which inhibits the leaching of phosphorus elements, and acid leaching can produce higher phosphorus content than deionized water leaching [71]. In addition, ettringite and C-S-H gel produced by a hydration reaction of cementing materials have good adsorption and solidification effects on phosphorus impurities. The leaching concentration of PCBAs is significantly affected by curing conditions. Open conditions, including standard and natural curing are more favorable to the solidification of phosphorus impurities than sealed and water curing. This is similar to previous findings [1,2].

## 4. Conclusions

Through a comparative study of the properties of PCBAs under different curing conditions, the following conclusions were obtained.

Water curing is beneficial to early strength development for the 7 d single aggregate strength reaching 5.26 MPa. For the single aggregate strength cured for 28 d, standard curing improved by 19.3% over others, including 292.6% over natural curing.

The curing conditions cause significant differences in the physical and mechanical properties of PCBAs by affecting the quantity, distribution and accumulation of hydration reaction products. Standard curing promotes the generation of ettringite and induces a shift towards more C-S-H gel with increased ratios of 29.71% and 42.75% over water and sealed-cured methods for 28 d, respectively. The hydration products tend to be generated in the interstices of gypsum particles and form a strong bond under standard conditions, while the others are distributed haphazardly, which is not as effective as the standard condition in consolidating the excess PG particles.

The isotherms of PCBAs under the four curing conditions suggest a slit-type pore structure. The PCBAs that are standard cured exhibit the maximum BET-specific surface area of 13.83 m^2^/g, suggesting more gel pore area. The water-cured PCBAs achieve the smallest average and most frequent pore size, 8.5 nm and 6.0 nm, but the natural-cured PCBAs exhibit a wide pore size distribution. The PCBAs achieved excellent solidification of phosphorus impurities compared to the original PG.

Comparing the overall physical and mechanical properties of PCBAs under the four curing conditions, water curing is recommended for its adaptability to the needs of industrial production with a shorter curing time.

The research in this paper provides an important reference for the industrial production and maintenance of phosphogypsum-based cold adhesive aggregates. However, due to the limitation of research time and conditions, the development law of PCBA performance under the combined curing mode has not been studied yet, and further research is expected in the future.

## Figures and Tables

**Table 1:** Chemical composition and physical properties of phosphogypsum, ordinary Portland cement and ground granulated blast-furnace slag (wt.%).

Compositions (wt%)	PG	GGBS	Cement
SiO_2_	8.34	29.57	21.75
Al_2_O_3_	1.27	14.02	6.45
Fe_2_O_3_	0.58	1.39	3.24
CaO	26.66	41.27	58.02
MgO	0.11	8.24	2.25
SO_3_	37.95	2.48	2.47
TiO_2_	0.13	0.95	0.51
P_2_O_5_	0.80	0.07	0.25
MnO	/	0.55	/
F	1.30	/	/
LOI	21.70	0.09	3.80
Physical properties			
Specific gravity	2.28	2.84	2.98
Specific surface area (m^2^/g)	0.50	1.38	1.79
D_10_ (μm)	6.09	1.52	1.18
D_50_ (μm)	33.87	10.87	10.59
D_90_ (μm)	100.57	27.85	33.78

**Table 2:** Characteristic parameters of the four curing conditions.

Sample Label	Curing Condition	Relative Humidity	Temperature
P8W	Water curing	100%	20 ± 1 °C
P8S	Sealed curing	<5%	20 ± 2 °C
P8N	Natural curing	>50%	20 ± 2 °C
P8M	Standard (moist) curing	>90%	20 ± 2 °C

**Table 3:** Cylinder compressive strength and softening coefficient at 28 d.

Sample Label	Cylinder Compressive Strength (Dry Status)/MPa	Cylinder Compressive Strength (Saturated Surface Dry Status)/MPa	Softening Coefficient
P8W	11.3	10.1	0.89
P8S	8.6	7.5	0.87
P8M	10.1	8.8	0.87
P8N	7.2	6.2	0.86

**Table 4:** Relative amounts of two kinds of water and carbonate contents.

Sample Label	Total Weight Loss (%)	Water in Dihydrate Gypsum (%)	Carbonates Content (%)	Water in Hydrations (%)
P8W	21.184	17.737	None	3.447
P8S	20.926	17.794	None	3.132
P8M	22.020	17.549	None	4.471
P8N	17.243	15.980	2.818	None

**Table 5:** Characterization of bands on the FTIR spectra.

Wavenumbers (cm^−1^)	Bands
525	Si-O out-of-plane bending vibration (δ_Si-O_) [53]
605, 670	S-O bending vibration [54]
875–878	CO_3_^2−^ out-of-plane bending vibration (v_2_) [55]
925	Si-O symmetry stretching vibration (v_3_) [53,56]
970	Si-O stretching vibration of Q^2^ (C-S-H) [57]
1003	Si-O stretching vibration of Q^3^ (C-S-H) [50]
1090	Si-O bending vibration in Q^3^ [52]
1100–1200	S-O symmetry stretching vibration of SO_4_ (v_3_) [54]
1417, 1470	C-O symmetry stretching vibration [52,56,58]
1685, 1624	H-O-H bending vibration [59]
3408	H-O-H stretching vibration [60]
3550–3554	H-O-H stretching vibration in gypsum [23]

**Table 6:** Specific surface area, pore volume and pore size of phosphogypsum-based cold-bonded aggregates.

Sample	S_BET_ (m^2^/g)	Pore Volume (cm^3^/g)	Average Pore Size (nm)	Most Frequent Pore Diameter (nm)
P8W	13.38	0.0486	8.5	6.0
P8S	9.78	0.0230	9.5	9.5
P8M	13.83	0.0513	8.9	8.9
P8N	5.66	0.0240	15.8	8.0

**Table 7:** Leaching results of P impurities (mg/L).

Sample	Acetic Acid Leaching (mg/L)	Static Leaching (mg/L)
PG	128.997	4.570
P8W	0.365	0.016
P8S	0.247	0.020
P8N	0.029	0.020
P8M	0.082	0.016

## Data Availability

The original contributions presented in this study are included in the article; further inquiries can be directed to the corresponding authors (due to privacy).
